# Dienophilic reactivity of 2-phosphaindolizines: a conceptual DFT investigation

**DOI:** 10.3762/bjoc.18.127

**Published:** 2022-09-13

**Authors:** Nosheen Beig, Aarti Peswani, Raj Kumar Bansal

**Affiliations:** 1 Department of Chemistry, The IIS (Deemed to be University), Jaipur 302020, India

**Keywords:** dienophilic reactivity, electronic chemical potential, electrophilicity index, Fukui function, global hardness, nucleophilicity index, 2-phosphaindolizines

## Abstract

The >C=P– or –N=P– functionality in 1,3-azaphospholo[1,5-*a*]pyridine, named as 2-phosphaindolizine and its 1- and 3-aza derivatives act as dienophiles and undergo Diels–Alder reactions with 1,3-dienes. However, the dienophilic reactivity is affected by the nature of the substituent groups on the two sides of the σ^2^,λ^3^-P atom and also by the presence of more nitrogen atom(s) in the five-membered ring. The conceptual density functional theory (DFT) calculations have been used in recent years to predict the reactivity of organic molecules in reactions. We calculated global hardness (η), global softness (S), electronic chemical potential (μ), electrophilicity (ω), and nucleophilicity (*N*) indices of four classes of 2-phosphaindolizines, on the basis of which their observed relative dienophilic reactivities could be rationalized. Besides, the Fukui functions of the carbon/nitrogen and phosphorus atoms of the >C=P– and –N=P– functionalities were also computed which revealed their hard electrophilic character and accorded well with the dienophilic reactivities observed experimentally. Furthermore, energies and symmetries of the lowest unoccupied molecular orbitals (LUMO) of 2-phosphaindolizines were found to be in conformity with their dienophilic reactivities.

## Introduction

**Figure 1:**
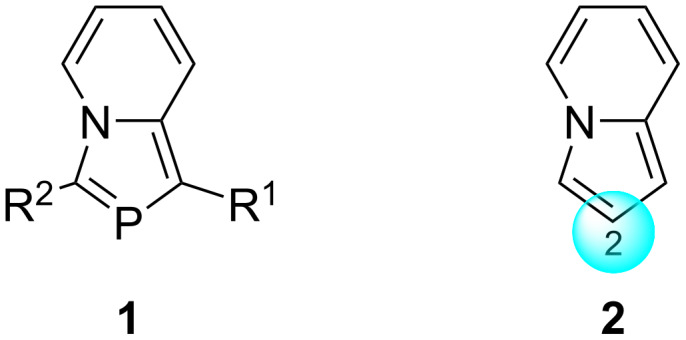
Structures of 2-phosphaindolizine (**1**) and indolizine (**2**).

In 1988, we developed a simple synthetic method for the synthesis of 1,3-azaphospholo[1,5-*a*]pyridine derivative (**1**, R^1^ = Me, R^2^ = PhCO) from the reaction of 2-ethyl-1-phenacylpyridinium bromide with PCl_3_ and Et_3_N, which was named as 2-phosphaindolizine perceiving it to result from a formal CH/P exchange at the 2-position of indolizine (**2**) (Figure 1) [1]. Subsequently a good library of these interesting compounds became accessible [1].

**Figure 2:**
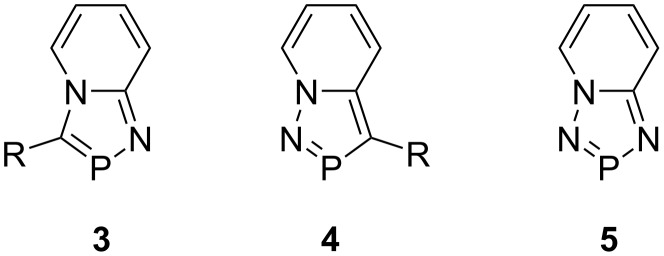
Structures of 1-aza-2-phosphaindolizines **3**, 3-aza-2-phosphaindolizines **4**, and 1,3-diaza-2-phosphaindolizine (**5**).

The methodology could be extended successfully to the synthesis of 1,3,4-diazaphospholo[1,2-*a*]pyridines, i.e., 1-aza-2-phosphaindolizines **3** [2], 1,2,3-diazaphospholo[1,5-*a*]pyridines, i.e., 3-aza-2-phosphaindolizines **4** [3], and 1,2,4,3-triazaphospholo[1,5-*a*]pyridine, i.e., 1,3-diaza-2-phosphaindolizine (**5**, Figure 2) [4]. We succeeded in developing another method involving a 1,5-electrocyclization of the initially formed pyridinium alkoxycarbonyl-dichlorophosphinomethylide followed by 1,2-elimination affording 1,3-bis(alkoxycarbonyl)-2-phosphaindolizines [5].

After having access to a good number of differently substituted derivatives of these four classes of 2-phosphaindolizines, we were motivated to explore their reactivity as they apparently have many active functionalities. In view of the earlier reported results of the Diels–Alders (DA) reaction across the >C=P– functionality in phosphaalkenes [6], phosphaketenes [6], heterophospholes [7], phosphinines [8], and azaphosphinines [9] (a recent review incorporates all these classes [10]), we investigated DA reactions across the >C=P– or –N=P– functionality present in these compounds. During this, we found that these compounds exhibited quite different dienophilic reactivities towards 2,3-dimethyl-1,3-butadiene (DMB). 2-Phosphaindolizines having electron-withdrawing groups (EWG) both at the 1 and 3-positions, namely 1,3-bis(ethoxycarbonyl)-1,3-azaphospholo[1,5-*a*]pyridine (**1**: R^1^ = R^2^ = COOEt) and its isoquinoline analogue, reacted with DMB and isoprene to give the [2 + 4] cycloadducts, in the latter case, regioselectively [11]. However, 2-phosphaindolizines having an EWG at the 3-position only, namely 3-ethoxycarbonyl-1-methyl-2-phosphaindolizine (**1**: R^1^ = Me, R^2^ = COOEt) did not undergo the DA reaction with DMB alone or in the presence of sulfur even when refluxing in toluene [12]. The reaction could be accomplished only in the presence of a Lewis acid catalyst, namely ethylaluminum dichloride [13]. Furthermore, when carrying out the reaction of compounds **1** (R^1^ = Me, R^2^ = COOMe, COOEt, COOCMe_3_) with DMB in the presence of the catalyst *O*-menthoxyaluminium dichloride, generated in situ, complete diastereoselectivity was observed.

The DA reactions of 1-aza-2-phosphaindolizines **3** with DMB and isoprene occurred at rt, although slowly and were speeded up by the use of sulfur or selenium which oxidized the phosphorus atom of the initially formed product thereby pushing the reaction in the forward direction [14].

**Figure 3:**
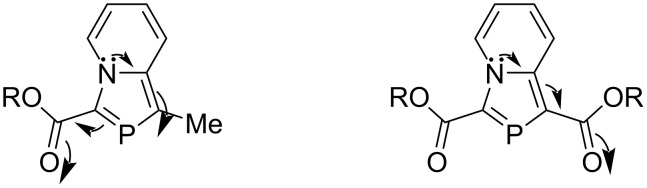
Transfer of the nitrogen lone-pair in 2-phosphaindolizines.

The difference in the reactivities of two classes of 2-phosphaindolizines namely the 2-phosphaindolizine substituted by the EWG at 3-position only (**1**, R^1^ = Me, R^2^ = CO_2_Me) and substituted by EWGs both at the 1- and 3- positions (**1**, R^1^ = R^2^ = CO_2_Me) could be rationalized on the basis of DFT calculations at the B3LYP/6-31G (d,p) level of theory wherein it was revealed that the nitrogen lone-pair is transferred effectively into the azaphospole ring. In compound **1** (R^1^ = Me, R^2^ = CO_2_Me) the EWG at the 3-position emphasizes this effect further. As a result, the >C=P– functionality becomes electron-rich and does not undergo a DA reaction with an electron-rich diene such as DMB. However, in the case of compound **1** (R^1^ = R^2^ = CO_2_Me), the EWG at the 1-position functions as electron-sink between the nitrogen lone-pair and the >C=P– functionality. As a result, the latter retains its electron-deficient character and undergoes DA reaction with DMB (Figure 3) [15].

The DFT based on the Hohenberg–Kohn theorems and later on the Kohn–Sham approximation made it possible to study the progress of organic reactions with manageable computational costs [16–17]. Parr and co-worker [18] developed “Conceptual DFT”, a subfield of DFT which allows to calculate various reactivity descriptors, such as electrochemical potential, electrophilicity and nucleophilicity indices, global hardness, electronegativity, etc.

The concept of hard–soft acid–base (HSAB) was used to explain the reactivity of the organic molecules towards electrophilic and nucleophilic reagents [19]. Thus a quantitative descriptor, the Fukui function was defined as


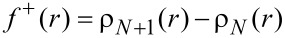


for nucleophilic attack and


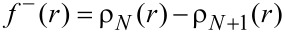


for electrophilic attack,

where ρ*_N_*_+1_(*r*), ρ*_N_*(r), and ρ_N−1_(*r*) are the electron densities at a point *r* in the system with *N*+1, *N*, and *N*−1 electrons, respectively, all with the ground-state geometry of the *N*-electron system. It was concluded that the regions of a molecule with a large Fukui function are chemically softer than the regions where the Fukui function is small. Thus, by invoking the HSAB principle it becomes possible to predict the behavior of a particular site in the molecule towards hard or soft reagents [19].

Yang and Mortier [20] suggested the use of the gross charge (*q*_r_) at a particular atom r in a molecule obtained from the Mulliken population analysis (MPA) for the calculation of the condensed Fukui function (*f*(r)) at that atom. The condensed Fukui function using MPA often has negative values and in this context, the use of Hirshfeld population analysis (HPA) based on the Stock-Holder idea was recommended [21–22].

We report herein different conceptual DFT descriptors of four classes of 2-phosphaindolizines and the attempts to compare the dienophilic reactivities of the >C=P– or –N=P– functionality present in these compounds towards 1,3-butadiene.

## Results and Discussion

We investigated the following model DA reactions (Table 1) at the DFT (B3LYP/6-31+G (d)) level of theory.

**Table 1:** Model DA reactions of 2-phosphaindolizines with 1,3-butadiene computed at the B3LYP/6-31+G(d) level of theory.

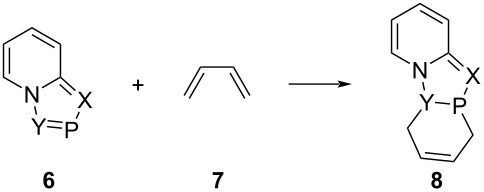									

**6**	**Aa**	**Ab**	**Ac**	**Ad**	**Ba**	**Bb**	**Ca**	**Cb**	**D**

X	CH	C-CO_2_Me	CH	C-CO_2_Me	N	N	CH	C-CO_2_Me	N
Y	CH	CH	C-CO_2_Me	C-CO_2_Me	CH	C-CO_2_Me	N	N	N

The values of the energies of the frontier molecular orbitals (FMOs) global hardness (η), global softness (S), electronic chemical potential (µ), electrophilicity (ω) and nucleophilicity (*N*) indices of 2-phosphaindolizines **6** and 1,3-butadiene (**7**) are given in Table 2.

**Table 2:** Energies of frontier molecular orbitals, global hardness, global softness, electronic chemical potential, electrophilicity, and nucleophilicity indices of 2-phosphaindolizines and 1,3-butadiene.

Compounds **6**	*E*_HOMO_ (eV)	*E*_LUMO_ (eV)	Global hardness η (eV)	Global softness S (eV)	Electronic chemical potential µ (eV)	Electrophilicity index ω (eV) × 10^2^	Nucleophilicity index^a^ *N* (eV) × 10^2^

**Aa**	−0.211	−0.025^b^	0.079	6.361	−0.132	0.110	–
**Ab**	−0.222	−0.033^b^	0.076	6.548	−0.146	0.139	–
**Ac**	−0.221	−0.068	0.077	6.510	−0.144	0.135	–
**Ad**	−0.230	−0.080	0.075	6.644	−0.155	0.160	–
**Ba**	−0.232	−0.001^c^	0.084	5.952	−0.148	0.128	–
**Bb**	−0.242	−0.080	0.081	6.184	−0.161	0.160	–
**Ca**	−0.231	−0.042^b^	0.085	5.899	−0.146	0.125	–
**Cb**	−0.240	−0.078	0.081	6.161	−0.159	0.156	–
**D**	−0.256	−0.052^b^	0.091	5.494	−0.164	0.148	–
**7**	−0.242	−0.042	0.100	5.000	−0.142	–	0.104

^a^With respect to TCNE = *E*_HOMO_ = −0.346; ^b^LUMO + 1 as LUMO is not of proper symmetry; ^c^LUMO + 3 as LUMO is not of proper symmetry.

2-Phosphaindolizines and 1,3-butadiene are soft electrophiles and nucleophile, respectively, and in accordance with the HSAB principle, they are expected to react. In the series **6A** of 2-phosphaindolizines, the softness decreases in the order **6Ad** > **6Ab** > **6Ac** > **6Aa**. No representatives of **6Aa** and **6Ab** have been prepared so far. Thus, compound **6Ad** is expected to undergo a DA reaction faster than compound **6Ac** which is in agreement with the reported results; the former reacts with DMB without the aid of a catalyst [11] whereas the latter undergoes DA reaction only in the presence of a catalyst [13]. However, it may be noted that although compounds **6Bb** and **6D** are less soft than **6Ac**, they undergo DA reaction without the aid of a catalyst [14]. As discussed later, this can be rationalized on the basis of the local hardness represented by the Fukui function.

The electronic chemical potential (µ) is another useful descriptor that reveals efficacy of charge transfer from the species of higher chemical potential to a species with lower chemical potential [23]. The reactivities of two substrates A and B with the same reagent C can be compared on the basis of the relative values of ∆μAC and ∆μBC; the greater the value of ∆µ is, the faster will be the reaction. In this context, it may be noted that except for compound **6Aa**, i.e., the unsubstituted 2-phosphaindolizine, the electronic chemical potentials of the other 2-phosphaindolizines are smaller than the electronic chemical potential of 1,3-butadiene indicating the possibility of an effective charge transfer from the latter to the former. Furthermore, the gap between the chemical potentials of 2-phosphaindolizines and 1,3-butadiene decreases in the order **6Ad** > **6Ab** > **6Ac**; **6Bb** > **6Ba**; **6Cb** > **6Ca**. This order is similar to the one derived on the basis of global hardness discussed earlier and is also in conformity with the experimental results.

The electrophilicity (ω) [24] and nucleophilicity (*N*) [25–26] indices are other useful descriptors that explain relative reactivities of molecules in chemical reactions [27]. The electrophilicity indices of 2-phosphaindolizines also decrease in the same order as observed on the basis of global hardness and electronic chemical potential. Furthermore, the electrophilicity index of compound **6D** (0.148) is close to that of **6Cb** (0.156) and like **6Cb**, it is expected to undergo a DA reaction with 1,3-butadiene without the aid of a catalyst, a fact in conformity with the experimental results.

### Concept of local hardness/softness

#### Fukui function analysis

As discussed earlier, the descriptor Fukui function was developed to determine the hard/soft character of the reactive site in a molecule [19–21]. The Fukui functions at the carbon/nitrogen and phosphorus atoms of the >C=P– or –N=P– functionality of 2-phosphaindolizines calculated from the Mulliken and Hirshfeld charges are given in Table 3.

**Table 3:** Fukui functions at the Y (C or N) and phosphorus atoms of the >Y=P– functionality of 2-phosphaindolizines (dienophile).

Compounds **6**	Fukui function *f*^+^(r) for nucleophilic attack		Fukui function *f*^−^(*r*) for electrophilic attack	
	Mulliken	Hirshfeld	Mulliken	Hirshfeld

**Aa**	C −0.086 P −0.276	C −0.162 P −0.186	–	–
**Ab**	C −0.019 P −0.269	C −0.042 P −0.184	–	–
**Ac**	C −0.206 P −0.130	C −0.039 P −0.124	–	–
**Ad**	C −0.154 P −0. 112	C −0.025 P −0.105	–	–
**Ba**	C −0.057 P −0.296	C −0.048 P −0.222	–	–
**Bb**	C −0.008 P −0.382	C −0.044 P −0.305	–	–
**Ca**	N −0.058 P −0.321	N −0.054 P −0.222	–	–
**Cb**	N −0.042 P −0.298	N −0.049 P −0.217	–	–
**D**	N(9) −0.047 P(8) −0.361	N(9) −0.052 P(8) −0.278	–	–
**7**	–	–	C(1) −0.166 C(4) −0.191	C(1) −0.186 C(4) −0.197

A close look at the Fukui functions calculated from the Mulliken and Hirshfeld charges reveals that they follow almost a similar pattern; in view of this, our further discussion is based on the Fukui functions calculated from the former. As the P atom is common in the >C=P– and –N=P– functionalities, we concentrated on the relative values of the Fukui functions of the P atom in different 2-phosphamdolizines. The values of the Fukui function of the P atom in the series of different 2-phosphaindolizines decrease in the order: **Ad** > **Ac** > **AB** > **Aa**; **Ba** > **Bb**; **Cb** > **Ca**.

Except the series, **Ba** > **Bb**, this order is similar to those obtained on the basis of the other descriptors discussed earlier. It may be noted that the values of the Fukui function of the P atom in compounds **D** (−0.361) and **Bb** (−0.382) are comparable with that in **Ca** indicating their comparable reactivities towards the DA reaction with 1,3-butadiene, i.e., they are expected to undergo the DA reaction without the aid of a catalyst.

### Frontier molecular orbital (FMO) treatment of the DA reaction

**Figure 4:**
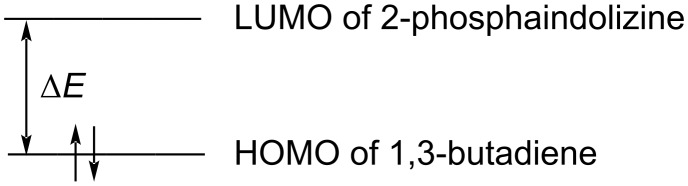
Energy gap (Δ*E*) between HOMO of 1,3-butadiene and LUMO of 2-phosphaindolizine.

The FMOs of a molecule are very important parameters to reveal its reactivity towards a reagent [28]. In the DA reaction of 2-phosphaindolizines with 1,3-butadiene, the HOMO of the latter will interact with the LUMO of the former and the energy gap (∆*E*) between the two will give an indication about the reactivity of the 2-phosphaindolizines (Figure 4).

**Figure 5:**
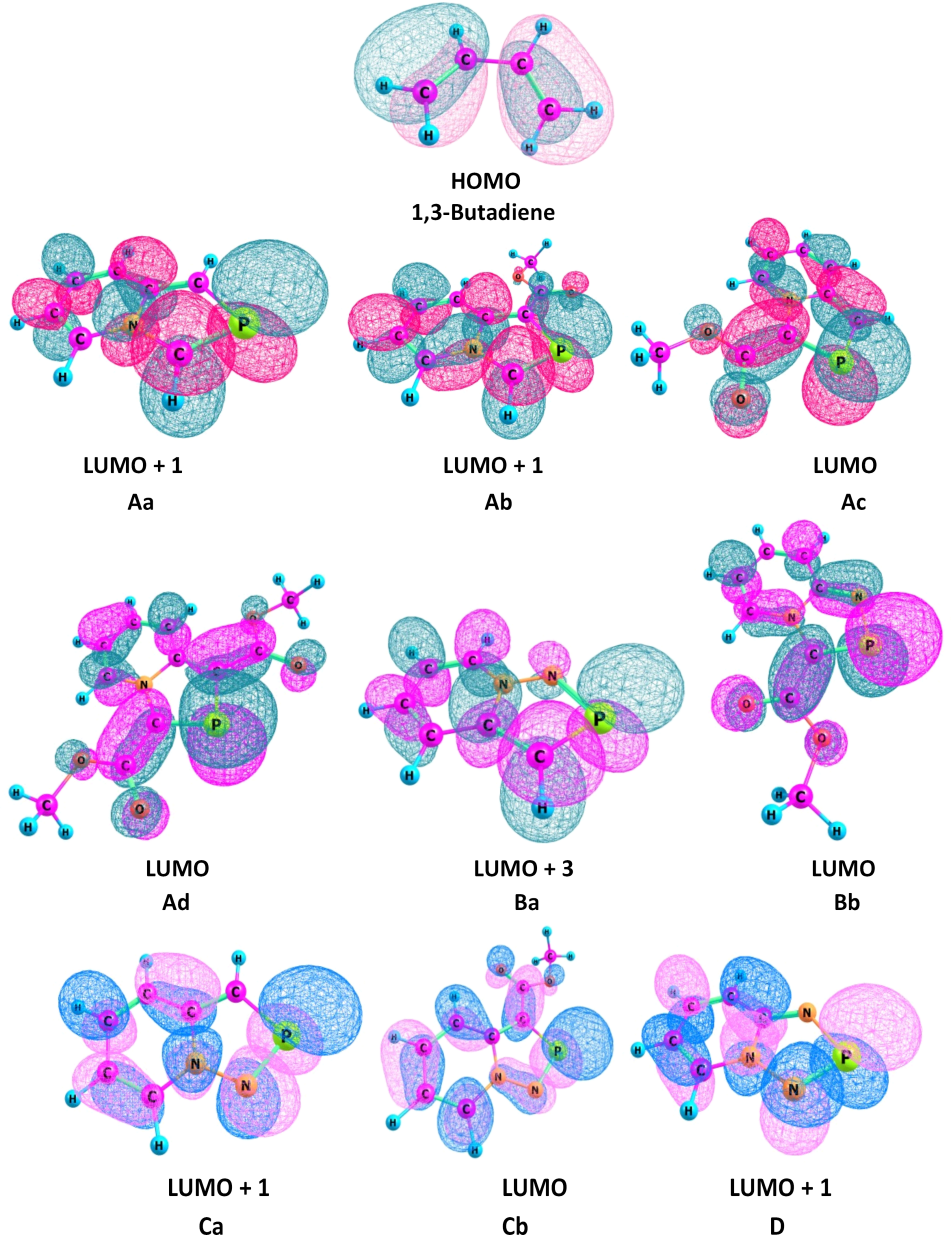
Kohn–Shan HOMO of 1,3-butadiene and LUMOs of 2-phosphaindolizines computed at the B3LYP/6-31+G(d) level of theory.

The HOMO of 1,3-butadiene (ψ_2_) and the LUMOs of different 2-phosphaindolizines are given in Figure 5. It may be mentioned that for compounds **Aa**, **Ab**, **Ba**, **Ca**, and **D**, the respective LUMOs were not found to be of the appropriate symmetry. Instead, LUMO + 1 for **Aa**, **Ab**, **Ca**, and **D** and the LUMO + 3 for **Ba** have the required symmetry.

A closer look at the energies of the LUMOs (or LUMO + 1 for **Aa**, **Ab**, **Ca**, **D** and LUMO + 3 for **Ba**) reveals that they increase in the following order: **Ad** < **Ac** < **Ab** < **Aa**. In view of this, the reactivity of 2-phosphaindolizines towards DA reaction with 1,3-butadiene is expected to change in the order: **Ad** > **Ac** > **Ab** > **Aa**. Similarly, the order of reactivity in the other two classes would be **Bb** > **Bc**; **Cb** > **Ca**. These orders of reactivities are almost similar to those obtained on the basis of other reactivity descriptors discussed earlier.

## Conclusion

The reactivity descriptors, namely global hardness/softness, electronic chemical potential, electrophilicity and nucleophilicity indices as well as the Fukui functions computed from the conceptual DFT calculation at the B3LYP/6-31+G (d) level of theory could be used successfully to rationalize the experimentally observed dienophilic reactivites of four classes of 2-phosphaindolizenes. The energies of the LUMOs (or in some cases LUMO + 1 or LUMO + 3) of 2-phosphaindolizines with respect to the energy of the HOMO of 1,3-butadiene were also found in accordance with their relative dienophilic reactivites. Thus, conceptual DFT descriptors can be advantageously used to predict the reactivities of the organophosphorus compounds.

## Computational Methods

All calculations were done using the *Gaussian 16* program [29]. We found that almost without exception, hybrid of Becke 3 and LYP correlation functional [30–31] has been used for determining reactivity descriptors [32–35] and the results were found to be independent of the basis sets [33]. In view of this, we carried out all calculations at the B3LYP/6-31+G (d) level of theory. Furthermore, Phukan et al. [32] calculated electrophilicity indices of a number of aziridines in the gas and solvent phases and observed that in both cases, a similar pattern of varying the values is followed. In view of this, we carried out all calculations in the gas phase only. Thus all geometries were optimized in the gas phase at the B3LYP/6-31+G(d) level of theory. Frequency calculations were done at the same level to determine zero-point correction and to characterize energy minimum with no imaginary frequency.

Chemical reactivity descriptors were calculated as follows:


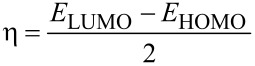



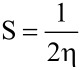



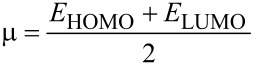



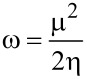



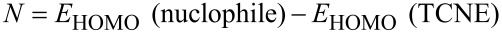



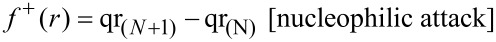



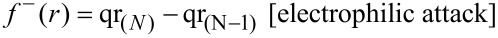


## Supporting Information

File 1 Cartesian coordinates of the geometries optimized (Table S1) at the B3LYP/6-31+G (d) level of theory.
