# Supplementary material for: Dienophilic reactivity of 2-phosphaindolizines: a conceptual DFT investigation
Source: Beilstein J Org Chem. 2022 Sep 13;18:1217–24. doi: 10.3762/bjoc.18.127 (PMC9490064; doi:10.3762/bjoc.18.127)
Supplement: File 1 — Cartesian coordinates of the geometries optimized (Table S1) at the B3LYP/6-31+G (d) level of theory. [file Beilstein_J_Org_Chem-18-1217-s001.pdf]

BEILSTEIN JOURNAL OF ORGANIC CHEMISTRY

Supporting Information

for

Dienophilic reactivity of 2-phosphaindolizines: a conceptual DFT investigation

Nosheen Beig, Aarti Peswani and Raj Kumar Bansal

Beilstein J. Org. Chem. **2022,** *18,* 1217–1224. doi:10.3762/bjoc.18.127

Cartesian coordinates of the geometries optimized (Table S1) at the B3LYP/6-31+G (d) level of theory

License and Terms: This is a supporting information file under the terms of the Creative Commons Attribution License (https://creativecommons.org/licenses/by/4.0). Please note that the reuse, redistribution and reproduction in particular requires that the author(s) and source are credited and that individual graphics may be subject to special legal provisions.
The license is subject to the *Beilstein Journal of Organic Chemistry* terms and conditions: (https://www.beilstein-journals.org/bjoc/terms)

SUPPORTING INFORMATION

TABLE 1 Cartesian coordinates of the optimized geometries

1,3-Butadiene

6	-1.549183489	-0.492897259	0.077638860
6	-0.728115488	0.553030384	-0.104873515
6	0.728127071	0.553033406	0.104877238
6	1.549180213	-0.492906623	-0.077640900
1	1.160286392	1.505783795	0.413862811
1	-1.160281995	1.505775381	-0.413867354
1	1.185701601	-1.452197280	-0.439297815
1	2.615414737	-0.414374326	0.116296987
1	-1.185756365	-1.452201275	0.439315654
1	-2.615414219	-0.414345753	-0.116320379

6Aa

6	1.135408817	1.461803535	0.000000000
6	2.377218971	0.901224866	0.000000000
6	2.523395259	-0.520771783	0.000000000
6	1.406181714	-1.313579209	0.000000000
6	0.099907209	-0.743634664	0.000000000
7	0.000000000	0.668016140	0.000000000
6	-1.155359429	-1.339702113	0.000000000

15	-2.466398535	-0.168064527	0.000000000
6	-1.296961626	1.113899673	0.000000000
1	3.246988557	1.549530126	0.000000000
1	3.515293349	-0.962149905	0.000000000
1	1.482677709	-2.396756290	0.000000000
1	0.955433005	2.530693343	0.000000000
1	-1.280110865	-2.416253477	0.000000000
1	-1.463049218	2.184349305	0.000000000

6Ab

6	0.000000000	0.629868440	0.000000000
7	-1.166205425	1.421176507	0.000000000
6	-1.105857665	2.801698877	0.000000000
6	0.095514903	3.445969712	0.000000000
6	1.300266824	2.686565074	0.000000000
6	1.248412740	1.314877689	0.000000000
6	-2.330070146	0.682508331	0.000000000
15	-2.080236624	-1.021034918	0.000000000
6	-0.326235752	-0.738148662	0.000000000
6	0.590203673	-1.882877720	0.000000000
8	0.228561831	-3.048813000	0.000000000
1	0.115510139	4.530593177	0.000000000

1	2.259333155	3.195733173	0.000000000
1	2.147555495	0.714152284	0.000000000
1	-2.064035910	3.308078757	0.000000000
1	-3.266243517	1.227637009	0.000000000
8	1.910340685	-1.539225606	0.000000000
6	2.835553505	-2.640908603	0.000000000
1	3.826683971	-2.184776462	0.000000000
1	2.695117668	-3.258569855	0.891038950
1	2.695117668	-3.258569855	-0.891038950

6Ac

6	1.085017173	-1.547463964	0.000000000
7	0.000000000	-0.646005700	0.000000000
6	-1.296496114	-1.134074486	0.000000000
6	-1.542122529	-2.478777584	0.000000000
6	-0.471172698	-3.412700502	0.000000000
6	0.816776210	-2.941892837	0.000000000
6	0.424327996	0.684390037	0.000000000
6	-0.418401421	1.881739459	0.000000000
8	0.041926357	3.012416728	0.000000000
6	2.318106994	-0.891873833	0.000000000
15	2.174097295	0.839368490	0.000000000

1	-2.574020694	-2.813990225	0.000000000
1	-0.673751310	-4.479331322	0.000000000
1	1.668397947	-3.615215270	0.000000000
1	-2.074132379	-0.387904263	0.000000000
1	3.251170586	-1.443441703	0.000000000
8	-1.764047719	1.655472126	0.000000000
6	-2.588375045	2.837592308	0.000000000
1	-3.615472482	2.470340546	0.000000000
1	-2.391321783	3.438156161	0.891432139
1	-2.391321783	3.438156161	-0.891432139

6Ad

6	-1.042284052	0.066797303	0.000000000
7	0.000000000	1.014881894	0.000000000
6	-0.285818671	2.367806363	0.000000000
6	-1.577558132	2.814802502	0.000000000
6	-2.650196907	1.890152817	0.000000000
6	-2.379515314	0.543702910	0.000000000
6	1.268254405	0.416393395	0.000000000
6	2.571875028	1.094501675	0.000000000
8	3.625612936	0.480456694	0.000000000
6	-0.543353638	-1.254581778	0.000000000

6	-1.306337810	-2.512639509	0.000000000
8	-0.788539216	-3.617216513	0.000000000
15	1.209376711	-1.324847351	0.000000000
1	-1.756471018	3.884765328	0.000000000
1	-3.677352808	2.242407034	0.000000000
1	-3.169474673	-0.194055084	0.000000000
1	0.570319552	3.022006918	0.000000000
8	-2.658006441	-2.351373314	0.000000000
8	2.534117194	2.456102042	0.000000000
6	3.819746150	3.109754639	0.000000000
1	3.597227345	4.177395574	0.000000000
1	4.386394065	2.830931856	0.891616002
1	4.386394065	2.830931856	-0.891616002
6	-3.426306840	-3.569414873	0.000000000
1	-4.469597540	-3.250934631	0.000000000
1	-3.202295363	-4.161157854	-0.891269385
1	-3.202295363	-4.161157854	0.891269385

6Ba

7	0.000000000	0.691286879	0.000000000
6	1.155512695	1.447786127	0.000000000
6	2.375636821	0.836144959	0.000000000

6	2.462840575	-0.587955601	0.000000000
6	1.321059601	-1.347264114	0.000000000
6	0.037925359	-0.727924772	0.000000000
7	-1.140226341	-1.322729568	0.000000000
15	-2.395963181	-0.191371566	0.000000000
6	-1.299613481	1.146186581	0.000000000
1	3.271633717	1.446626319	0.000000000
1	3.438484732	-1.064928081	0.000000000
1	1.344438858	-2.431511811	0.000000000
1	1.017971188	2.522988083	0.000000000
1	-1.471665769	2.215658733	0.000000000

6Bb

6	-1.497640561	-1.129655105	0.000000000
7	-0.132539212	-0.765602865	0.000000000
6	0.863680221	-1.722256851	0.000000000
6	0.533601335	-3.050536530	0.000000000
6	-0.826800933	-3.457753727	0.000000000
6	-1.821740390	-2.511113706	0.000000000
6	0.000000000	0.622458402	0.000000000
15	-1.585403936	1.356905184	0.000000000
7	-2.362711286	-0.120733612	0.000000000

1	1.333594990	-3.783268613	0.000000000
1	-1.075380887	-4.514980088	0.000000000
1	-2.874901675	-2.768878251	0.000000000
1	1.874172235	-1.338858917	0.000000000
6	1.304389676	1.281080847	0.000000000
8	1.156754399	2.626992680	0.000000000
8	2.400154185	0.733974565	0.000000000
6	2.371183964	3.397285770	0.000000000
1	2.047921018	4.438445976	0.000000000
1	2.963549157	3.176762497	0.892148179
1	2.963549157	3.176762497	-0.892148179

6Ca

7	0.000000000	-0.636390025	0.000000000
6	1.138916368	-1.408876761	0.000000000
6	2.369691001	-0.814436780	0.000000000
6	2.476972560	0.606258297	0.000000000
6	1.341259164	1.377201740	0.000000000
6	0.055984072	0.766476806	0.000000000
6	-1.235995893	1.294766847	0.000000000
15	-2.398118281	-0.004986169	0.000000000
7	-1.219615311	-1.196731623	0.000000000

1	3.256289095	-1.439122331	0.000000000
1	3.457451768	1.072917599	0.000000000
1	1.392975080	2.461451479	0.000000000
1	0.957780790	-2.476234998	0.000000000
1	-1.436378949	2.359291457	0.000000000

6Cb

6	-0.015280176	-0.783462523	0.000000000
7	-1.335439252	-1.239140227	0.000000000
6	-1.647939066	-2.576792641	0.000000000
6	-0.647787858	-3.509888407	0.000000000
6	0.710411810	-3.091140205	0.000000000
6	1.022740583	-1.751698103	0.000000000
7	-2.310312266	-0.304820754	0.000000000
15	-1.652699248	1.223933398	0.000000000
6	0.000000000	0.625405846	0.000000000
1	-0.908176836	-4.562843339	0.000000000
1	1.502593422	-3.833922555	0.000000000
1	2.043944992	-1.390859646	0.000000000
1	-2.709181723	-2.790263330	0.000000000
6	1.232203031	1.417755535	0.000000000
8	0.965079848	2.748228389	0.000000000

8	2.370974462	0.973241804	0.000000000
6	2.106563790	3.622266795	0.000000000
1	1.694553369	4.631885995	0.000000000
1	2.716554485	3.454144728	0.891802494
1	2.716554485	3.454144728	-0.891802494

6D

7	0.000000000	0.661506117	0.000000000
6	1.156920455	1.398832231	0.000000000
6	2.364722683	0.754050248	0.000000000
6	2.416004737	-0.667555306	0.000000000
6	1.256496531	-1.406184551	0.000000000
6	-0.002099445	-0.748734990	0.000000000
7	-1.221362549	-1.275662252	0.000000000
15	-2.330449497	-0.026297918	0.000000000
7	-1.225330092	1.222037059	0.000000000
1	3.275681552	1.342433917	0.000000000
1	3.380007254	-1.167729487	0.000000000
1	1.257335759	-2.490477079	0.000000000
1	1.018296648	2.472629163	0.000000000
